# ﻿A new species of *Eudigraphis* (Diplopoda, Polyxenida, Polyxenidae) from East China, with embryonic and post-embryonic development observations, and mitogenomic and genetic divergence analyses

**DOI:** 10.3897/zookeys.1247.155348

**Published:** 2025-07-22

**Authors:** Si-Qi Yang, Yun Bu, Nerivania Nunes Godeiro, Yan Gao, Ya-Li Jin

**Affiliations:** 1 Shanghai Natural History Museum, Shanghai Science & Technology Museum, Shanghai 200041, China Shanghai Natural History Museum, Shanghai Science & Technology Museum Shanghai China

**Keywords:** Bristle millipedes, mitochondrial genome, molecular analysis, taxonomy, stadium

## Abstract

*Eudigraphishuadongensis* Yang & Bu, **sp. nov.** from east China is described and illustrated. The new species is compared with other congeners in detail and observations about its embryonic and post-embryonic development are provided. The complete mitochondrial genome of the new species is also analyzed, which represents the first published mitogenome of Polyxenida. The genetic divergence between *E.huadongensis***sp. nov.** and its congeners was analyzed using Neighbor-Joining inference based on COI gene sequences. *Eudigraphishuadongensis***sp. nov.** clustered with *E.nigricans* and *E.kinutensis*, supporting the morphological identification. The newly assembled mitogenome is 15,206 bp in length and its gene order is unique, possibly representing a pattern among Polyxenida species.

## ﻿Introduction

Polyxenida Verhoeff, 1934, known as bristle millipedes, is a group of tiny diplopods with approximately 190 species recorded in the world (Wang et al. 2025). The Chinese Polyxenida is poorly studied with only 12 species reported (Yang and Bu 2021; Wang et al. 2025). *Eudigraphis* Silvestri, 1948 is a well-defined genus of Polyxenidae Lucas, 1840 distributed in China and Japan. It is characterized by eight ocelli on each side of the head; trunk with ten tergites, nine pleural projections, a telson, and 13 pairs of legs; sixth and seventh antennal articles with three and two bacilliform sensilla, respectively; posterior edge of the tergites with a single continuous row of trichomes; caudal trichomes with numerous barbed hooks arranged in a line, and a mandible without a molar tuft. Six species of *Eudigraphis* are known worldwide: *E.takakuwai* (Miyosi, 1947), *E.nigricans* (Miyosi, 1947), and *E.kinutensis* (Haga, 1950) from Japan, *E.sinensis* Ishii & Liang, 1990, *E.taiwaniensis* Ishii, 1990, and *E.xishuangbanna* Ishii & Yin, 2000 from China (Miyosi 1947; Takashima and Haga 1950; Ishii 1990; Ishii and Liang 1990; Ishii and Yin 2000; Karasawa et al. 2020). *Eudigraphisnigricans* was also reported in Zhejiang Province of China recently (Wang et al. 2025) and in Jiangsu Province in the sea-side forest (personal observation).

During our soil fauna investigation in East China, plenty of bristle millipedes were obtained from Jiangsu, Shanghai, and Zhejiang. Among them, one species of the genus *Eudigraphis* was identified as new to science and it is described in the present paper. The embryonic and post-embryonic development of the new species was observed. The complete mitochondrial genome of the new species was also sequenced and analyzed, the first to be available for the order Polyxenida. The genetic divergence among species of the genus were analyzed using mitochondrial cytochrome c oxidase subunit 1 gene (COI).

## ﻿Materials and methods

### ﻿Sample collection

**Figure 1.**
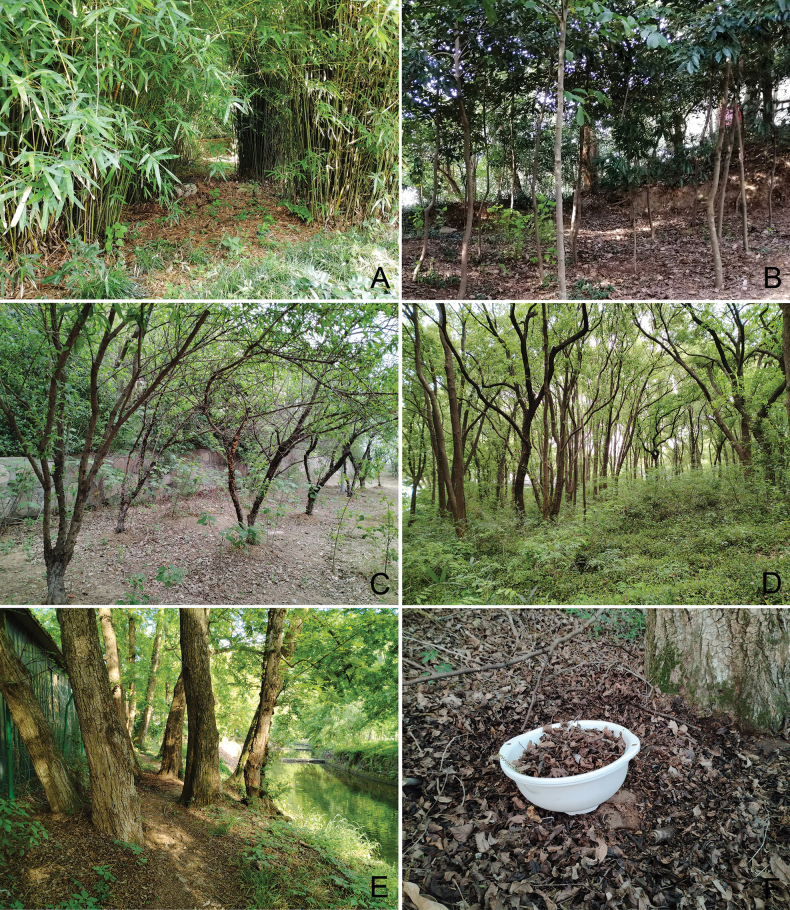
The habitats of *Eudigraphishuadongensis* sp. nov. **A.** Daji Mountain, Jiangsu; **B.** Tianma Mountain, Shanghai; **C.** Shoutaohu Park, Jiangsu; **D.** Lingyan Mountain, Jiangsu; **E.** Huzhou city, Zhejiang; **F.** Collection in the field.

Most of the specimens were collected from leaf litter under broad-leaf forest or bamboo forest using an entomological aspirator. Some were extracted from soil and litter samples by using Berlese-Tullgren funnels. Specimens were kept in absolute ethanol and frozen. Their habitats are shown in Fig. 1A–F.

### ﻿Morphological study

Specimens were mounted on slides using Hoyer’s solution and dried in an oven at 50 °C. Morphological observations were performed under a phase contrast microscope (Leica DM 2500). Photographs were taken with a digital camera installed on the microscope (Leica DMC 4500). Specimens were measured from head to telson, excluding the caudal bundle of trichomes. The sex of specimens was identified by the presence of sex organs between the coxae of the second pair of legs. The specimens were prepared for taxonomic illustration following the technique of Short and Huynh (2010a) with minor modifications. The leg was described following the naming scheme of Short and Huynh (2010b). For scanning electron microscopy observation, specimens were dehydrated in solutions of increasing concentration of ethanol and examined directly using FEI QUANTA650 scanning electron microscopy. All specimens are deposited in the collection of the Shanghai Natural History Museum (**SNHM**), Shanghai, China.

Abbreviations used in the descriptions: **b**–trichome socket b on the dorsal side of the telson, **BT**–branched comb teeth, **c**–conical sensillum, **c1, 2, 3**–trichome sockets on the dorsal side of the telson, **CT**–simple comb teeth, **FL**–fimbriate lamella, **IL**–intermediate lobe, **IS**–intermediate sensillum, **LP**–lateral palp of gnathochilarium, **MC**–molar comb, **MP**–medial palp of the gnathochilarium, **MPr**–molar process, **s**–setiform sensillum, **SaO**–salivary ostiole, **SL**–serrate limb, **SmL**–smooth limb, **SNHM**–Shanghai Natural History Museum, **Ta**–anterior thick sensillum, **Ti**–intermediate thick sensillum, **Tp**–posterior thick sensillum.

### ﻿Molecular experiments

The specimens used for the experiment were collected from several localities in east China from 2017 to 2023. Samples were preserved in absolute ethanol at -20 °C for DNA extraction. Prior to DNA extraction, each specimen was observed under a stereomicroscope to confirm the species identification. For whole genome sequencing, both specimens from Jiangsu and Shanghai preserved in alcohol were sent to Shanghai Yao’en Biotechnology Co., Ltd, China, where all laboratory procedures, including DNA extraction and library construction were made following custom procedures. DNA was extracted from a single individual of each specimen using the TIANamp MicroDNA extraction kit (Tiangen Co., Ltd, China). Libraries were constructed using KAPA Hyper Prep Kit (Roche). An Illumina NovaSeq platform was used to produce paired-end reads with 150 bp length. Approximately 10 Gb of data from each specimen was generated and used to assemble the mitogenomes. For COI gene fragments, total genomic DNA was extracted from one specimen with Promega genomic DNA purification kit following the manufacturer’s instructions. The primer pair LCO (5’-GGTCAACAAATCATAAAGATATTGG-3’), HCO (5’-TAAACTTCAGGGTGACCAAAAAATCA-3’) (Folmer et al. 1994) was used for amplification and sequencing.

### ﻿Mitogenome assembly and annotation

Using BBTools (sourceforge.net/projects/bbmap/), with the “clumpify.sh” and “bbduk.sh” pipelines, we analyzed the raw sequencing data to remove sequencing adapters, remove reads with low quality, remove contaminants and correct potential errors. The trimmed data were then analyzed using MitoZ v. 3.6 (Meng et al. 2019) integrated tools as follows: MEGAHIT v. 1.2.9 (Li et al. 2015) for assembly; Tiara v. 1.0.1 (Karlicki et al. 2022) and HMMER v. 3.4 (Wheeler and Eddy 2013) for homology searches and sequence alignment. For annotation, BLAST+ (Gertz et al. 2006), GeneWise (Birney et al. 2004), Infernal v. 1.1.5 (Nawrocki and Eddy 2013), and MiTFi v. 0.1 (Jühling et al. 2012). Visualization of the results of annotation and coverage graphic was done using Circos v. 0.69 (Krzywinski et al. 2009), BWA v. 0.7.17 (Li and Durbin 2009), and SAMtools v. 1.18 (Li et al. 2009). After publication, mitogenome sequences and raw sequencing data will be available in NCBI at (https://www.ncbi.nlm.nih.gov/) under the accession numbers PV243313 and PV243314, respectively. Bioproject number: PRJNA1228998. After annotation we observed that the mitogenomes of both specimens from Jiangsu and Shanghai of *Eudigraphishuadongensis* sp. nov. were identical, confirming the morphological analyses.

### ﻿Genetic divergence analysis

In order to analyze genetic divergences among species of *Eudigraphis*, 11 DNA barcodes from five populations of the new species were sequenced. 18 COI gene sequences of Polyxenida and two sequences of the families Lophoproctidae Silvestri, 1897 and Sphaerotheriidae Brandt, 1833 (outgroup) were downloaded from GenBank and analyzed. The detailed information and accession numbers of all sequences analyzed in this study are listed in Table 1. To infer the position of the new species described, a Neighbor-Joining tree was constructed based on COI gene sequences by MEGA X (Kumar et al. 2018) with the Jukes-Cantor model (Jukes and Cantor 1969) and 1000 bootstrap replicates. The genetic distance (K2P-distance) was calculated using MEGA X (Kimura 1980; Kumar et al. 2018).

**Table 1.** Taxonomic information, collection site, size, and GenBank accession number of the partial or complete sequences of COI of the species used in the analysis.

Species and voucher	Family	Location	Length (bp)	GenBank Number	Reference
*Eudigraphishuadongensis* sp. nov. JS-WX-BY2021006	Polyxenidae	China: Jiangsu	1536	PV243313	present study
*Eudigraphishuadongensis* sp. nov. SH-TMS-BY2021005	Polyxenidae	China: Shanghai	1536	PV243314	present study
*Eudigraphishuadongensis* sp. nov. SH-TMS-BY2021005	Polyxenidae	China: Shanghai	658	PV189309	present study
*Eudigraphishuadongensis* sp. nov. SH-TMS-BY2021006	Polyxenidae	China: Shanghai	658	PV189310	present study
*Eudigraphishuadongensis* sp. nov. SH-TMS-BY2021007	Polyxenidae	China: Shanghai	658	PV189311	present study
*Eudigraphishuadongensis* sp. nov. SH-TMS-BY2021008	Polyxenidae	China: Shanghai	658	PV189312	present study
*Eudigraphishuadongensis* sp. nov. SH-TMS-BY2021009	Polyxenidae	China: Shanghai	658	PV189313	present study
*Eudigraphishuadongensis* sp. nov. WX-DJS-BY2021001	Polyxenidae	China: Jiangsu	658	PV189314	present study
*Eudigraphishuadongensis* sp. nov. WX-DJS-BY2021002	Polyxenidae	China: Jiangsu	658	PV189315	present study
*Eudigraphishuadongensis* sp. nov. WX-DJS-BY2021004	Polyxenidae	China: Jiangsu	658	PV189316	present study
*Eudigraphishuadongensis* sp. nov. ZJ-HZ-BY2023004	Polyxenidae	China: Zhejiang	658	PV189317	present study
*Eudigraphishuadongensis* sp. nov. SZ-LYS-BY2023022	Polyxenidae	China: Jiangsu	658	PV189318	present study
*Eudigraphishuadongensis* sp. nov. SZ-STH-BY2023003	Polyxenidae	China: Jiangsu	658	PV189319	present study
*Eudigraphisnigricans* CZHZS3	Polyxenidae	China: Zhejiang	633	PQ141065	Wang et al. 2025
*Eudigraphisnigricans* E001	Polyxenidae	Japan	602	LC010874	Niikura 2014 (Unpublished)
*Eudigraphissinensis* CZHZS1	Polyxenidae	China: Zhejiang	646	PQ142931	Wang et al. 2025
*Eudigraphissinensis* CZNJS1	Polyxenidae	China: Jiangsu	647	PQ142932	Wang et al. 2025
*Polyxenushangzhoensis* CZCZS1	Polyxenidae	China: Anhui	666	PQ142930	Wang et al. 2025
*Eudigraphisnigricans* Di41	Polyxenidae	Japan	534	LC456731	Karasawa et al. 2020
*Eudigraphisnigricans* Di1	Polyxenidae	Japan	534	LC456732	Karasawa et al. 2020
*Eudigraphisnigricans* Di60	Polyxenidae	Japan	534	LC456750	Karasawa et al. 2020
*Eudigraphiskinutensis* Di38	Polyxenidae	Japan	534	LC456729	Karasawa et al. 2020
*Eudigraphiskinutensis* Di51	Polyxenidae	Japan	534	LC456744	Karasawa et al. 2020
*Eudigraphiskinutensis* Di16	Polyxenidae	Japan	534	LC456735	Karasawa et al. 2020
*Eudigraphistakakuwai* Di57	Polyxenidae	Japan	534	LC456748	Karasawa et al. 2020
*Eudigraphistakakuwai* Di58	Polyxenidae	Japan	534	LC456749	Karasawa et al. 2020
*Eudigraphistakakuwai* Di36	Polyxenidae	Japan	534	LC456727	Karasawa et al. 2020
*Eudigraphistakakuwai* Di43	Polyxenidae	Japan	534	LC456738	Karasawa et al. 2020
*Eudigraphistakakuwai* Di48	Polyxenidae	Japan	534	LC456742	Karasawa et al. 2020
*Eudigraphistakakuwai* Di8	Polyxenidae	Japan	534	LC456733	Karasawa et al. 2020
*Eudigraphistakakuwai* Di45	Polyxenidae	Japan	534	LC456740	Karasawa et al. 2020
*Lophoturussineprecessus* CZYNS1	Lophoproctidae	China: Yunnan	625	PQ142933	Wang et al. 2025
Sphaerotheriidae sp. HYS-2012	Sphaerotheriidae	China: Zhejiang	1536	NC_018361	Dong et al. 2012

## ﻿Results

### ﻿Taxonomic account

**Class Diplopoda de Blainville in Gervais 1844**

**Subclass Penicillata Latreille, 1831**

**Order Polyxenida Verhoeff, 1934**

**Family Polyxenidae Lucas, 1840**

**Subfamily Monographinae Condé, 2008**

#### ﻿Genus Eudigraphis

Taxon classificationAnimaliaPolyxenidaPolyxenidae

Silvestri, 1948

C7AF2B34-428F-51DE-8CA4-8E2E1FEA6161

##### Type species.

*Eudigraphisjaponica* Silvestri, 1948 (= *Eudigraphistakakuwai*); type locality: Ehime-ken and Kanagawa of Japan.

##### Diagnosis.

Head with eight ommatidia on each side. Body with ten segments, nine pleural projections, a telson, and 13 pairs of legs. Antenna with eight articles, the sixth antennal article has three long bacilliform sensilla, some species also have one conical sensillum and one setiform sensillum; the seventh antennal article has two long bacilliform sensilla, some species also have one conical sensillum and one setiform sensillum; eighth antennal article is shorter than article VII, with four finger-shaped sensory cones. Tergites with two lateral clusters of trichomes plus a single continuous row of trichomes close to posterior edge. Caudal trichomes on telson arranged as a penicil of hooked and barbate trichomes. The mandible has a comb-lobe, an intermediate plate, and a proximal plate, without a molar tuft.

##### Distribution.

China (Jiangsu, Shanghai, Taiwan, Yunnan, Zhejiang), Japan.

#### ﻿ Eudigraphis huadongensis

Taxon classificationAnimaliaPolyxenidaPolyxenidae

Yang & Bu sp. nov.

151F673C-248A-588E-89EF-6B346E8E1B4B

https://zoobank.org/A656A9F7-3484-4849-A065-2D601F5577C6

Figs 2 , 3 , 4 , 5 , 6 , 7 , 8 , 9 , 10 , 11 , 12 , 13 , Tables 1 , 2 , 3 , 4 , 5 , 6

##### Material examined.

***Holotype***: • female (slide no. JS-WX-PX2022012) (SNHM), China, Jiangsu Province, Wuxi, Daji Mountain, alt. 5 m, 31°32’N, 120°12’E, 2022-X-26, coll. Y. Bu. ***Paratypes*** (30 females, 11 males): • 1 female (slide no. JS-WX-PX2021001), 1 male (slide no. JS-WX-PX2021002), ibidem, 2021-VII-8, coll. Y. Bu; • 11 females (slides no. JS-WX-PX2022003–JS-WX-PX2022008, JS-WX-PX2022010–JS-WX-PX2022011, JS-WX-PX2022013–JS-WX-PX2022015), 1 male (slide no. JS-WX-PX2022009), ibidem, 2022-X-26, coll. Y. Bu; • 2 females (slides no. SH-TMS-PX2020001, SH-TMS-PX2020002), China, Shanghai, Tianma Mountain, alt. 99 m, 31°4’N,121°9’E, 2020-VII-31, coll. S. Q. Yang, Y. L. Jin & Y. Bu; • 3 female (slides no. SH-TMS-PX2021002, SH-TMS-PX2021003, SH-TMS-PX2021005), 1 male (slide no. SH-TMS-PX20210004), ibidem, 2021-IX-25, coll. Y. L. Jin, S. Q. Yang, Godeiro N. N. & Y. Bu; • 6 females (slides no. JS-SZ-PX2023004–JS-SZ-PX2023005, JS-SZ-PX2023007–JS-SZ-PX2023008, JS-SZ-PX2023013–JS-SZ-PX2023014), 6 males (slides no. JS-SZ-PX2023003, JS-SZ-PX2023006, JS-SZ-PX2023009–JS-SZ-PX2023012), Jiangsu Province, Suzhou, Shoutaohu Park, alt. 7 m, 31°17’N, 120°31’E, 2023-IV-14, coll. Y. Gao & Y. Bu; • 5 females (slides no. JS-SZ-PX2023023–JS-SZ-PX2023027), Jiangsu Province, Suzhou, Lingyan Mountain, alt. 30 m, 31°16’N, 120°31’E, 2023-IV-15, coll. Y. Gao & Y. Bu; • 2 females (slides no. ZJ-HZ-PX2023004–ZJ-HZ-PX2023005), 2 males (slides no. ZJ-HZ-PX2023009–ZJ-HZ-PX2023010), Zhejiang Province, Huzhou, Changxing, alt. 211 m, 31°1’N, 119°47’E, 2023-IV-30, coll. Y. Gao & Y. Bu.

**Figure 2.**
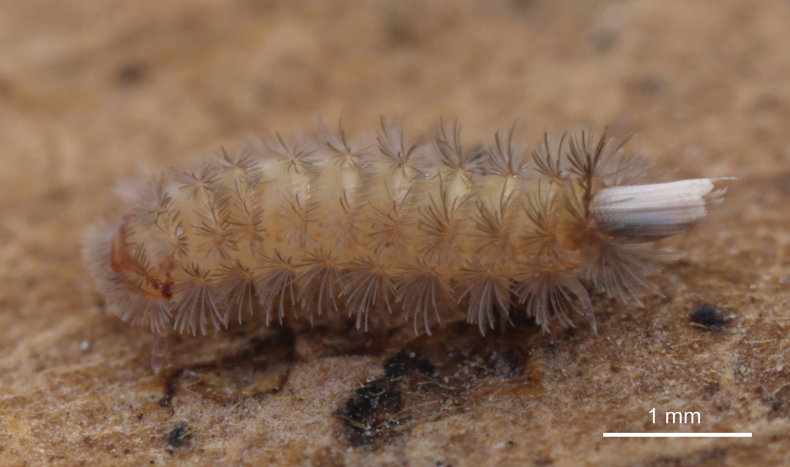
Live specimen of *Eudigraphishuadongensis* sp. nov.

##### Additional specimens examined.

***Stadium I***: • 7 individuals (slides no. SH-TMS-2020004–SH-TMS-20200010), China, Shanghai, Tianma Mountain, alt. 99 m, 31°4’N, 121°9’E, 2020-VII-31, coll. Y. L. Jin & S. Q. Yang. ***Stadium II***: • 3 individuals (slides no. JS-WX-PX2018023–JS-WX-PX2018025), China, Jiangsu Province, Wuxi, Daji Mountain, alt. 5 m, 31°32’N, 120°12’E, 2018-X-8, coll. Y. Bu; • 1 individual (slide no. JS-TMS-PX2018036), China, Shanghai, Tianma Mountain, alt. 99 m, 31°4’N,121°9’E, 2020-VII-31, coll. Y. Bu; • 2 individuals (slides no. JS-WX-PX2021005, JS-WX-PX2021008), ibidem, 2021-VII-8, coll. Y. Bu; ***Stadium III***: • 6 individuals (slides no. JS-WX-PX2021014–JS-WX-PX2021019), China, Jiangsu Province, Wuxi, Daji Mountain, alt. 5 m, 31°32’N, 120°12’E, 2021-IX-3, coll. Y. Bu. ***Stadium IV***: • 5 individuals (slides no. JS-WX-PX2018014–JS-WX-PX2018018), China, Jiangsu Province, Wuxi, Daji Mountain, alt. 5 m, 31°32’N, 120°12’E, 2018-X-8, coll. Y. Bu; • 1 individual (slide no. JS-WX-PX2021011), ibidem, 2021-IX-3, coll. Y. Bu. ***Stadium V***: • 6 individuals (slides no. JS-WX-PX2018006–JS-WX-PX2018011), China, Jiangsu Province, Wuxi, Daji Mountain, alt. 5 m, 31°32’N, 120°12’E, 2018-X-8, coll. Y. Bu. ***Stadium VI***: • 3 individuals (slides no. JS-WX-PX2018004, JS-WX-PX2018005, JS-WX-PX2018012), China, Jiangsu Province, Wuxi, Daji Mountain, alt. 5 m, 31°32’N, 120°12’E, 2018-X-8, coll. Y. Bu. ***Stadium VII***: • 2 females (slides no. JS-WX-PX2018001, JS-WX-PX2018003), 1 male (slide no. JS-WX-PX2018002), China, Jiangsu Province, Wuxi, Daji Mountain, alt. 5 m, 31°32’N, 120°12’E, 2018-X-8, coll. Y. Bu; • 1 female (slide no. SH-XSS-PX2017001), China, Shanghai, Sheshan Mountain, alt. 100 m, 31°6’ N, 121°12’E, 2017-V-10, coll. Y. Bu & Y. L. Jin.

##### Diagnosis.

*Eudigraphishuadongensis* sp. nov. is characterized by three long bacilliform sensilla, one conical sensillum and one setiform sensillum on the sixth antennal article, two long bacilliform sensilla, one conical sensillum and one setiform sensillum on seventh antennal article; posterior vertex trichome groups with 11–16 sockets in anterior row and 7–13 sockets in posterior row; 12 slender sensilla on lateral palp of gnathochilarium and 20 or 21 conical sensilla on the medial palp; 2+2 lamellae and 5+5 clypeo-labral setae on the labrum. The mandible has 14 branched comb teeth, ten rows of simple comb teeth, three longitudinal groups of intermediate sensilla, 14 serrate limbs, one smooth limb, 13 salivary ostioles, seven molar processes and seven molar combs. The leg setae have a coniform pleated base. The telson has 11–17 ornamental trichomes on each side dorsal to the caudal bundle; hooked caudal trichomes with a maximum of eight hooks.

##### Description.

Adult with 13 pairs of legs, body length 3.4 mm on average (3.0–3.6 mm, *n* = 45, holotype 3.4 mm), caudal bundle 0.4–0.5 mm (Fig. 2).

***Coloration*.** Body evenly yellowish brown dorsally, pale yellow ventrally. Head with one dark brown transverse band on vertex connecting with red-brown eyes. Antenna with articles IV–VIII dark brown, with the basal three articles milky white. Legs variable in color, with last four or five podomere sections pale purple, basal sections white or purple. Body trichomes yellow-brown and caudal bundle trichomes grey to brown (Figs 2, 9).

**Figure 3.**
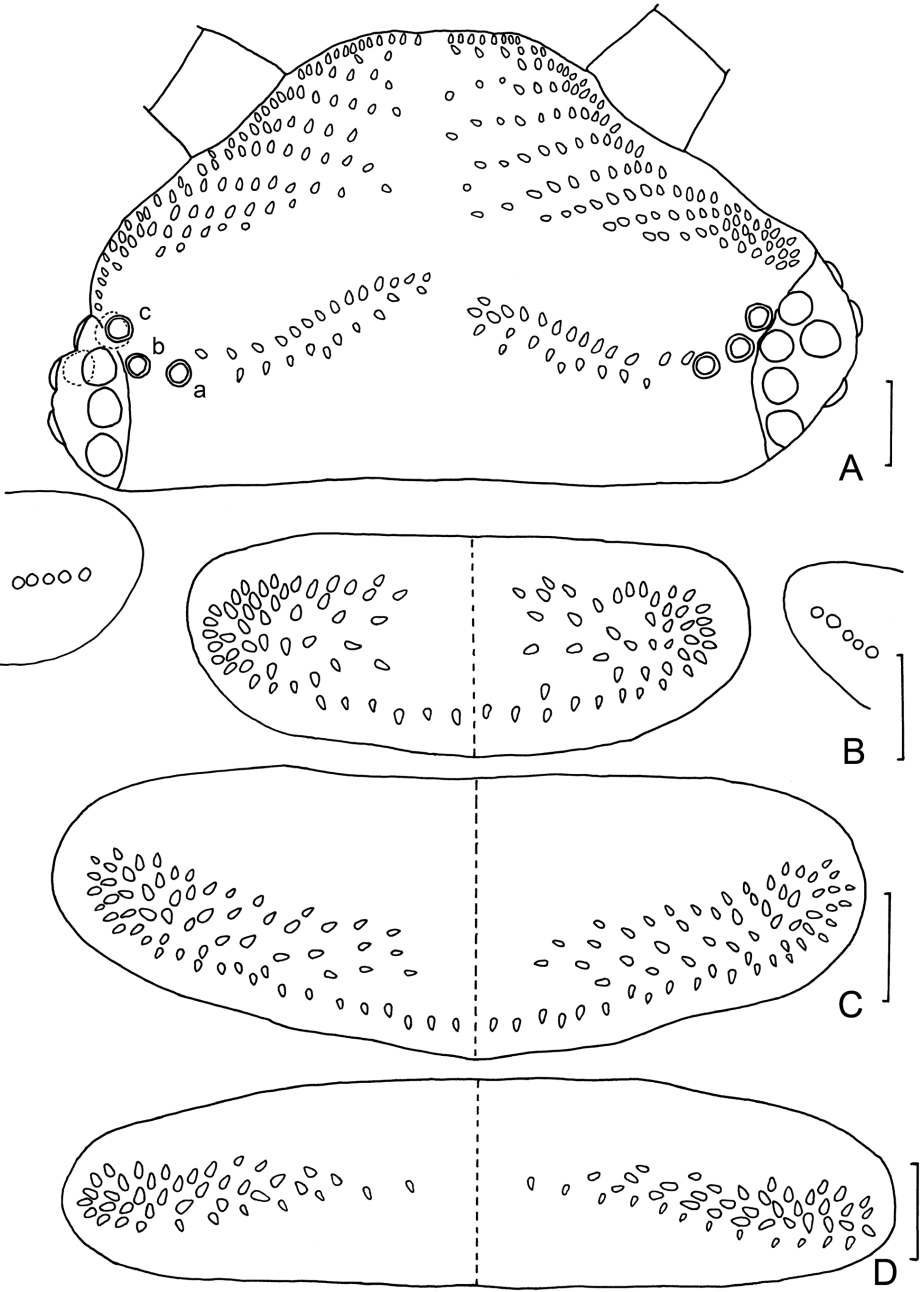
*Eudigraphishuadongensis* sp. nov. Holotype. **A.** Head; **B.** Collum; **C.** Tergite II showing pattern of trichome insertions; **D.** Tergite X. Abbreviations: a, b, c trichobothria a, b, c. Scale bars: 50 μm.

***Head*** length 314 μm on average (270–450 μm, *n* = 45), width 500 μm on average (400–600 μm, *n* = 45) (Fig. 5A), each side with eight ommatidia: five dorsal and three lateral ones (Figs 3A, 5B, 7H). Vertex with two posterior trichome groups separated by a large medial gap, each group arranged in two close adjacent rows, anterior row with large trichome sockets, posterior row with small trichome sockets (Figs 3A, 5A, 5G, 5H). Holotype has 14 sockets on both sides of anterior row, and ten sockets on both sides of posterior row (Figs 3A, 5G, 5H). Paratypes with 11–16 sockets in anterior row and 7–13 in posterior row on each side.

**Figure 4.**
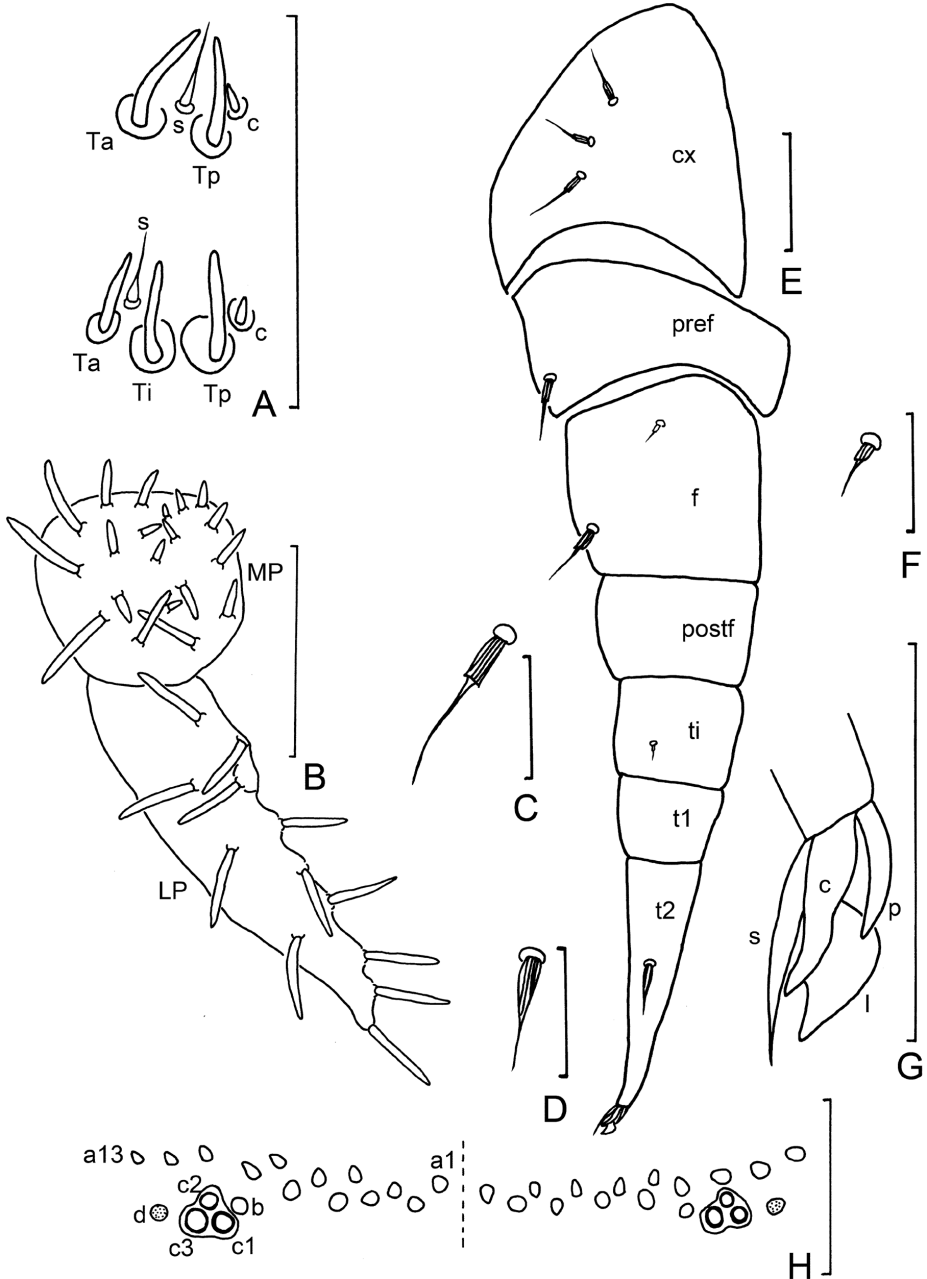
*Eudigraphishuadongensis* sp. nov. Holotype. **A.** Sensilla on antennal articles VI and VII; **B.** Gnathochilarium (MP–medial palp; LP–lateral palp); **C.** Typical setae of coxa, prefemur, and femur; **D.** Pointed seta on tarsus II; **E.** Leg 3, right side (cx–coxa; pref–prefemur; f–femur; postf–post-femur; ti–tibia; t1, 2–tarsus 1, 2); **F.** Short seta on femur and tibia; **G.** Telotarsus-claw (s–setiform process; c–claw; p–posterior lateral process; l–lamella process); **H.** Pattern of insertions of dorso-medial trichomes on telson. Abbreviations: c–conical sensillum; s–setiform sensillum;Ta–thick anterior sensillum; Ti–thick intermediate sensillum; Tp–thick posterior sensillum. Scale bars: 50 μm (**A, B, E, H**); 20 μm (**C, D, F, G**).

**Figure 5.**
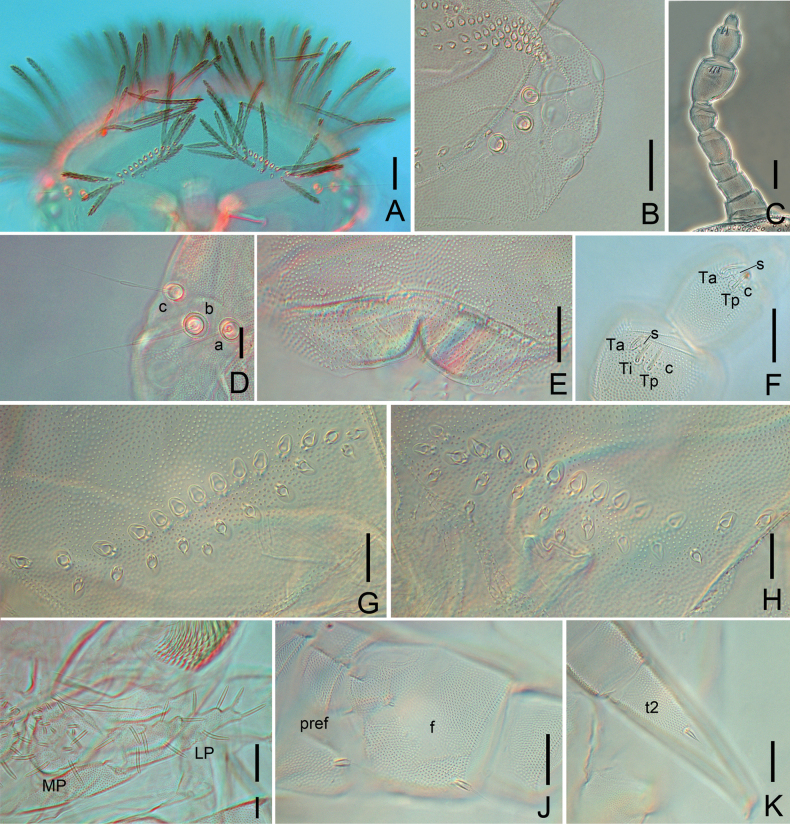
*Eudigraphishuadongensis* sp. nov. **A.** Head, dorsal view; **B.** Ommatidia of right eye; **C.** Left antenna; **D.** Trichobothria a, b, c on head, left side; **E.** Labrum; **F.** Antennal articles VI and VII; **G.** Posterior vertex trichome sockets, left side; **H.** Posterior vertex trichome sockets, right side; **I.** Gnathochilarium; **J.** Prefemur (pref) and femur (f) of leg 5, showing setae; **K.** Tarsus 2 (t2). Abbreviations: LP–lateral palp; MP–medial palp; c–conical sensillum; s–setiform sensillum; a–thick anterior sensillum; Ti–thick intermediate sensillum; Tp–thick posterior sensillum. Scale bars: 50 μm (**A, C**); 20 μm (**B, D–K**).

***Antennae*** length 420 μm on average (400–500 μm, *n* = 45), consist of eight antennal articles, article VIII shorter than VII (Fig. 5C). Antennal article VI with three thick bacilliform sensilla: anterior Ta (10–16 μm), intermediate Ti (11–17 μm) and posterior Tp (11–17 μm), one setiform sensillum s (7–9 μm) between Ta and Ti, and one conical sensillum c (2–3 μm) close to Tp (Figs 4A, 5F, 7I). Antennal article VII with two thick bacilliform sensilla Ta (11–17 μm) and Tp (10–16 μm), one setiform sensillum s (5–7 μm) between them, and one conical sensillum c (2–3 μm) close to Tp (Figs 4A, 5F, 7I). Antennal article VIII with four sensory cones (5–8 μm).

***Trichobothria*.** Trichobothrium a (posterior position), trichobothrium b (lateral position), and trichobothrium c (anterior position) with narrow cylindrical funicles, with sockets of equal size, forming an isosceles triangle with equal distance between ab and bc (Figs 3A, 5D, 7H).

***Labrum*.** Surface heavily covered by coarse granules, anterior margin with 2+2 lamellae on each side of the median cleft, clypeo-labrum with 5+5 short setae, 18–27 μm (Fig. 5E).

***Gnathochilarium*.** Lateral palp (90–107 μm) 2.1–2.3 times as long as medial palp (40–50 μm), each medial palp (MP) with 20 conical or slender sensilla, lateral palps (LP) each with 12 conical sensilla (Figs 4B, 5I).

**Figure 6.**
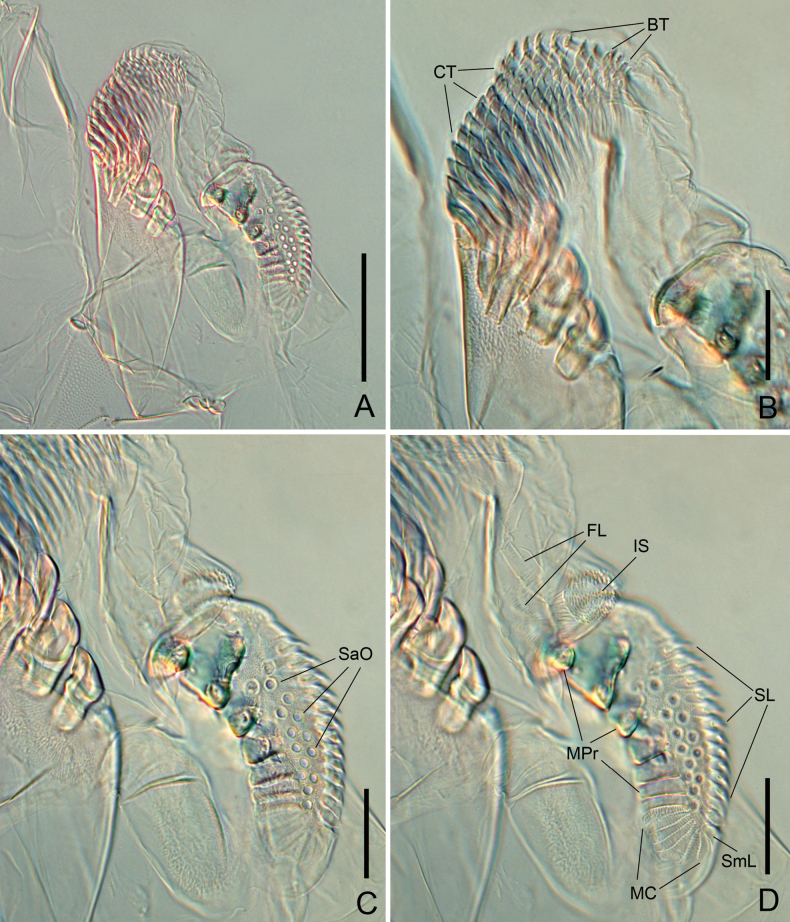
*Eudigraphishuadongensis* sp. nov. mandible. **A.** Overall view; **B.** Comb-lobe; **C.** Proximal plate showing 13 salivary ostioles (SaO); **D.** Intermediate lobe and proximal plate showing fine structures Abbreviations: FL–fimbriate lamella; IS–intermediate sensillum; MC–molar comb; MPr–molar process; SL–serrate limb; SmL–smooth limb. Scale bars: 50 μm.

***Mandible*.** Comb-lobe with a row of 14 branched comb teeth (BT) and ten rows of simple comb teeth (CT) (Fig. 6A, B); intermediate plate with intermediate lobe (IL), intermediate sensilla (IS) pointed at apex and arranged in three longitudinal groups, and several fimbriate lamellae (FL) deeply incised into long pointed processes (Fig. 6D); proximal plate bearing molar plate (Fig. 6C, D) consisting of 14 broad serrated limbs (SL) with stoutly chitinous spines at apices and one smooth limb (SmL), wide granulated zone, 13 salivary ostioles (SaO) arranged roughly in two rows (Fig. 6C), seven molar processes (MPr) and seven molar combs (MC) with regularly arranged teeth.

***Trunk*** composed of ten tergites, nine pleural projections and telson (Fig. 2). Collum with trichome sockets arranged in two oval-shaped groups in lateral positions opposite each other, connected by a single posterior row of trichome sockets in the middle (Figs 3B, 7A), with 50+48 trichome sockets in holotype (Figs 3B, 7D), and five trichome sockets on each lateral protuberance in holotype (Figs 3B, 7A), with 86–103 in total and five or six on lateral protuberance each side in paratypes (Table 2). Tergite II with 62+61 trichomes in holotype (Figs 3C, 7B), 103–132 in total in paratypes connected by a continuous posterior row of trichomes. Tergites II–X exhibit a consistent pattern of trichome insertions. Tergite X with fewer trichomes than other tergites (Figs 3D, 7C), 76 in holotype and 64–110 in paratypes. The number of trichomes for each tergite is given in Table 2.

**Table 2.** Number of trichome sockets on tergites of *Eudigraphishuadongensis* sp. nov. (holotype in brackets).

Tergites	Number of sockets (*n* = 45)
I	86–103 (98)
II	103–132 (123)
III	103–124 (119)
IV	109–132 (114)
V	120–144 (129)
VI	110–151 (134)
VII	112–145 (124)
VIII	104–144 (126)
IX	94–132 (108)
X	64–110 (76)

**Figure 7.**
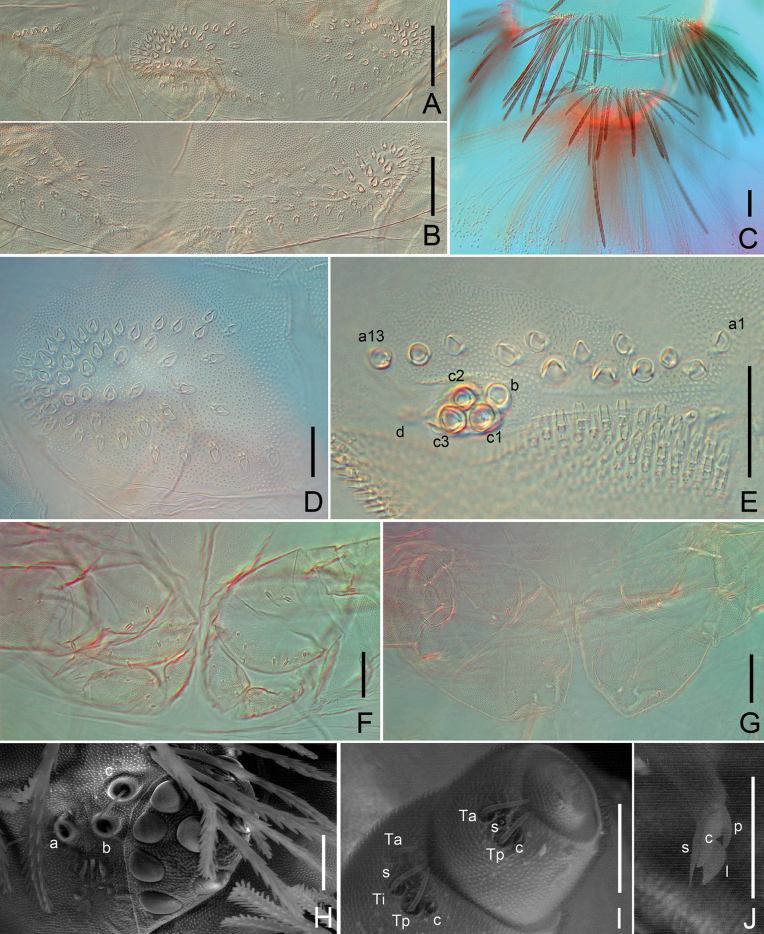
*Eudigraphishuadongensis* sp. nov. **A.** Collum; **B.** Tergite II; **C.** Tergite; **X.** And telson, showing trichomes; **D.** Trichome sockets of collum, left side; **E.** Ornamental trichome sockets of telson, left side; **F.** Female genital vulvae; **G.** Male penis; **H.** Eye and trichobothria under scanning electronic microscope; **I.** Antennal articles VI and VII; **J.** Telotarsus-claw. Abbreviations: c–claw; l–lamella process; p–posterior lateral process; s–setiform process. Scale bars: 50 μm (**A–C, F–G**); 20 μm (**D**–**E, H–J**).

***Legs*.** Leg 1 lacks tarsus 1. Chaetotaxy (setae on each leg article) (Table 3): coxa 1: one seta, coxa 2: two setae, coxae 3–13: 0–4 setae; prefemur with one seta; femur usually with one large, long seta and one short seta in similar shape (Fig. 4E, F), short seta sometimes absent on legs 1 and 13; tibia with one short seta (Fig. 4E, F), occasionally absent on legs 11–13; post-femur and tarsus 1 without seta, and tarsus 2 with one pointed seta. Large and long setae on legs with coniform pleated base (Figs 4C, 4E, 5J). Pointed setae on tarsus 2 with acute triangular base (Figs 4D, 4E, 5K).

**Table 3.** Leg chaetotaxy of *Eudigraphishuadongensis* sp. nov. (holotype in brackets).

Legs	Podomeres						
	coxa	prefemur	femur	post-femur	tibia	tarsus 1	tarsus 2
1	1 (1) ^a^	1 (1) ^a^	1(1) ^a^	-	1 (1) ^c^	/	1 (1) ^d^
2	2 (2)	1 (1)	2(2) ^b^	-	1 (1)	-	1 (1)
3	2–3 (3)	1 (1)	2(2)	-	1 (1)	-	1 (1)
4	2–4 (4)	1 (1)	2(2)	-	1 (1)	-	1 (1)
5	2–4 (2)	1 (1)	2(2)	-	1 (1)	-	1 (1)
6	2–3 (3)	1 (1)	2(2)	-	1 (1)	-	1 (1)
7	2–3 (2)	1 (1)	2(2)	-	1 (1)	-	1 (1)
8	2–3 (2)	1 (1)	2(2)	-	1 (1)	-	1 (1)
9	2–3 (2)	1 (1)	2(2)	-	1 (1)	-	1 (1)
10	2 (2)	1 (1)	2(2)	-	1 (1)	-	1 (1)
11	1–2 (1)	1 (1)	2(2)	-	0–1 (0)	-	1 (1)
12	1–2 (1)	1 (1)	2(2)	-	0–1 (0)	-	1 (1)
13	0–2 (0)	1 (1)	1–2 (2)	-	0–1 (0)	-	1 (1)

Notes. ^a^ large seta; ^b^ 1 large seta and 1 short seta; ^c^ short seta; ^d^ pointed seta.

Telotarsus short, with a robust and pointed claw, narrow posterior lateral process, equal to half length of claw, anterior setiform process longer than claw, and a large, triangular lamella (Figs 4G, 7J).

***Sex organ*.** Female with paired genital vulvae between coxa of leg 2, each consists of three plates: one large upper plate with six short setae, two small lower plates each with five setae (Fig. 7F). Male with paired penes between coxa 2 and two pairs of coxal glands on coxal plates of legs 8 and 9, each penis with eight short setae (Fig. 7G). All short setae on sex organs similar to the larger leg setae.

***Telson*.** Ornamental trichomes arranged either side dorsal to the caudal bundle (Fig. 7C), with 13+13 trichomes ‘a’ (holotype) (Figs 4H, 7E), and 11–17 trichomes ‘a’ on each side in paratypes (some asymmetry present). All have a single trichome ‘b’ and three trichome ‘c’ with large protruding base sockets c1, c2 and c3 arranged in a triangle (Figs 4H, 7E). Circular indentation d present (Figs 4H, 7E).

***Caudal bundles*** composed of a bundle of uniform, long hooked trichomes (Fig. 7C). Females with two latero-sternal bundles of trichome sockets of nest trichomes. Males without nest trichomes, but with two groups of large trichome sockets ventrally. Hooked trichomes with a maximum of eight hooks.

##### Variation.

Among 52 adults observed, the number of trichomes on head, tergites, and telson are fairly variable and usually asymmetrical. The posterior of the vertex varied with 11–16 sockets in anterior row and 7–13 in posterior row. The ornamental trichomes showed asymmetry between sides: 12+14 in one specimen, 13+12 in two specimens, 15+14 in four specimens, 16+17 in two specimens and 13+16 in one specimen. The clypeo-labrum with 6+6 setae was observed in two specimens, 5+4 setae in five specimens, 4+4 setae in five specimens. Medial palp gnathochilarium with 21+21 conical sensilla was observed in five specimens. On the female genital vulvae, 7–11 setae were observed on the upper plate in ten specimens instead of more common six setae, and six setae (instead of five) on one of the lower plates in five specimens.

##### Etymology.

The species was named after the Chinese words “Huadong” = “East China” which is the region where all type specimens were collected.

##### Remarks.

*Eudigraphishuadongensis* sp. nov. is similar to *E.sinensis*, *E.xishuangbanna*, and *E.taiwaniensis* in having the same number of sensilla on antennal articles VI and VII, similar body color, and same number of sensilla on the medial palp of gnathochilarium. They can be distinguished by the number of sensilla on the lateral palp of gnathochilarium (12 in *E.huadongensis* sp. nov. and *E.taiwaniensis* vs 13 in *E.sinensis*, 17 in *E.xishuangbanna*), mandible structures (one smooth limb on proximal plate in *E.huadongensis* sp. nov. vs two or three in others), labrum (with 2+2 lamellae in *E.huadongensis* sp. nov. and *E.taiwaniensis* vs 3+3 in *E.sinensis*, 3+3 or 4+4 in *E.xishuangbanna*), shape of large seta on legs (coniform base without other affiliated structures in *E.huadongensis* sp. nov. vs oval base with spines, pubescence, or processes in *Eudigraphis* species), and number of hooks on caudal trichome (maximum eight in *E.huadongensis* sp. nov. vs maximum four or five in others). All seven species of the genus *Eudigraphis* are compared in Table 4.

**Table 4.** Comparison of world species of genus *Eudigraphis*. Abbreviations used are explained in Materials and methods.

Characters		*E.huadongensis* sp. nov.	*E.sinensis*	*E.xishuangbanna*	*E.taiwaniensis*	*E.takakuwai*	*E.nigricans*	*E.kinutensis*
**Distribution**		China (Jiangsu, Shanghai, Zhejiang)	China (Jiangsu, Zhejiang)	China (Yunnan)	China (Taiwan)	Japan	China (Zhejiang, Jiangsu); Japan	Japan
**Body length (mm)**		3.0–3.6	2.4–2.8	3.5–4.0	2.9–3.3	3.8–4.5	3.0–4.2	2.5–3.0
**Coloration**		evenly yellowish brown	evenly yellowish brown	?	body yellowish brown	body cream yellow	head black, body dorsally cream yellow with two rows of blackish brown stripes	body gray
**Habitats**		forest floor	under the bark of trees	under the bark of trees	forest floor	forest floor or under the bark of trees	sea cliffs in Japan; under the bark of a tree or in the litter of forests in China.	under the bark of trees
**Sensilla on antenna**	**article VI**	Ta, s, Ti, Tp, c	Ta, s, Ti, Tp, c	Ta, s, Ti, Tp, c	Ta, s, Ti, Tp, c	Ta, Ti, Tp	Ta, Ti, Tp	Ta, Ti, Tp
	**article VII**	Ta, s, Tp, c	Ta, s, Tp, c	Ta, s, Tp, c	Ta, s, Tp, c	Ta, Tp	Ta, Tp	Ta, Tp
**Mandible**	**comb lobe**	14 BT and 10 rows of CT	11–12 BT and 6 rows of CT	15 BT and 8 rows of CT	14–15 BT and 10 rows of CT	16 BT and 10 rows of CT	13 BT and 8 rows of CT	13 BT and 7 rows of CT
	**arrangement of IS**	3 longitudinal groups	3 longitudinal groups	3 longitudinal groups	3 longitudinal groups	3 longitudinal groups	3 longitudinal groups	3 longitudinal groups
	**number of SL**	14	15–18	14	16–18	16	17	13
	**number of SmL**	1	2	3	2	3	2	5
	**number of SaO**	13	10–13	9	12–16	27	13	14
	**number of MPr**	7	8	7	7	7	8	8
	**number of MC**	7	5–7	6	8	9	5	6
**Labrum**	**number of lamellae**	2+2	3+3	4+4 (3+3)	2+2	?	3+3	?
	**clypeo-labral setae**	5+5	6+6	5+5 (7+7)	4+4	5+5	5+5 (6+6)	7
**Number of sockets on posterior vertex**	**anterior row**	11–16	11–13	13–19	20–27	10	20	20
	**posterior row**	7–13	7–9	5–9	8–9	?	10	8
**Number of sensilla on Gnathochilarium**	**medial palp**	20–21	21	21	21	10	10	?
	**lateral palp**	12	13	17	12	12	12	7
**Number of dorsal ornamental trichomes a**	**female**	11–17	9	14	15	?	5	?
	**male**	11–13	5–6	5–6	12–13	?	4	?
**Shape of large setae on legs**		base coniform pleated	base oval, pleated with distal long spines	base oval, pleated with pubescence	base oval, pleated with two distal acute spinal processes	?	base coniform, smooth	?
**Number of hooks on caudal trichome**		3–8	3–4	2–5	3–4	3–4	3–6	3–5

### ﻿Embryonic development observation

**Figure 8.**
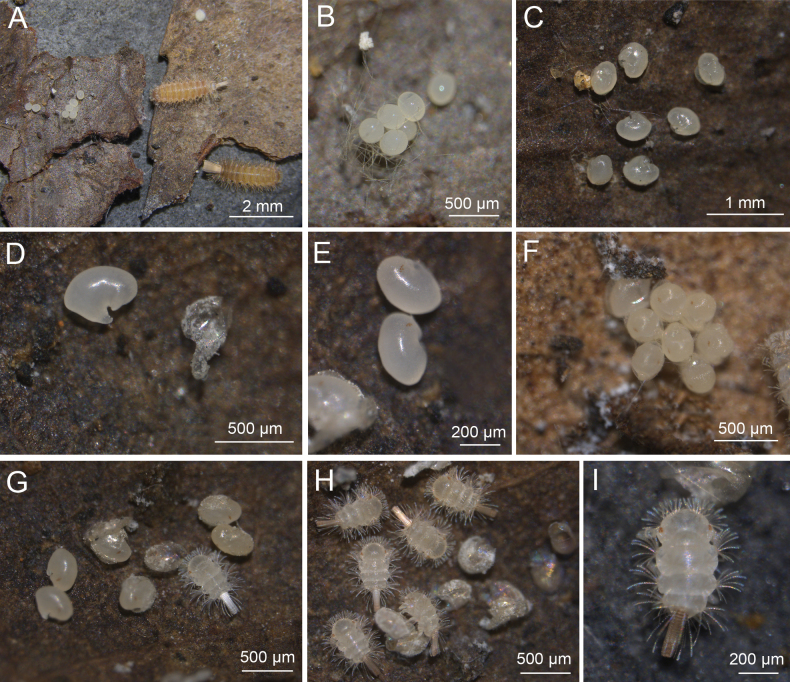
Embryonic development of *Eudigraphishuadongensis* sp. nov. **A.** Adults and eggs; **B.** Eggs protected by caudal trichomes; **C, D.** Embryo of two weeks; **E.** Embryos with ommatidia pigments present; **F.** Embryos observed in the fourth week; **G.** Embryos and new-hatched juvenile; **H.** Juveniles of stadium I after 2 days; **I.** Juvenile of stadium I, showing brown caudal bundle.

The living specimens of *E.huadongensis* sp. nov. from Jiangsu and Zhejiang were raised in the lab, and their embryonic development was observed and recorded. The females laid eggs in clusters and protected them with caudal trichomes (Fig. 8A, B). The eggs are oval, white, 350 μm in length and 240 μm in width, and they became transparent after one week (Fig. 8B). The buds of antenna and tail were apparently seen after two weeks, after the egg-shell splits and releasing the pupoid embryo (Fig. 8C, D). The pigments of the five ommatidia appeared in the third week (Fig. 8E, F). The embryonic development was completed in 30 days at room temperature. The new-hatched juveniles were white at the beginning (Fig. 8G) and became pale brown after one or two days, the caudal bundle also turned brown (Fig. 8H, I). In general, the process of the embryonic development of *E.huadongensis* sp. nov. is similar to *Monographisqueenslandicus* Huynh & Veenstra, 2013 observed by Huynh and Veenstra (2014).

### ﻿Post-embryonic development observation

**Figure 9.**
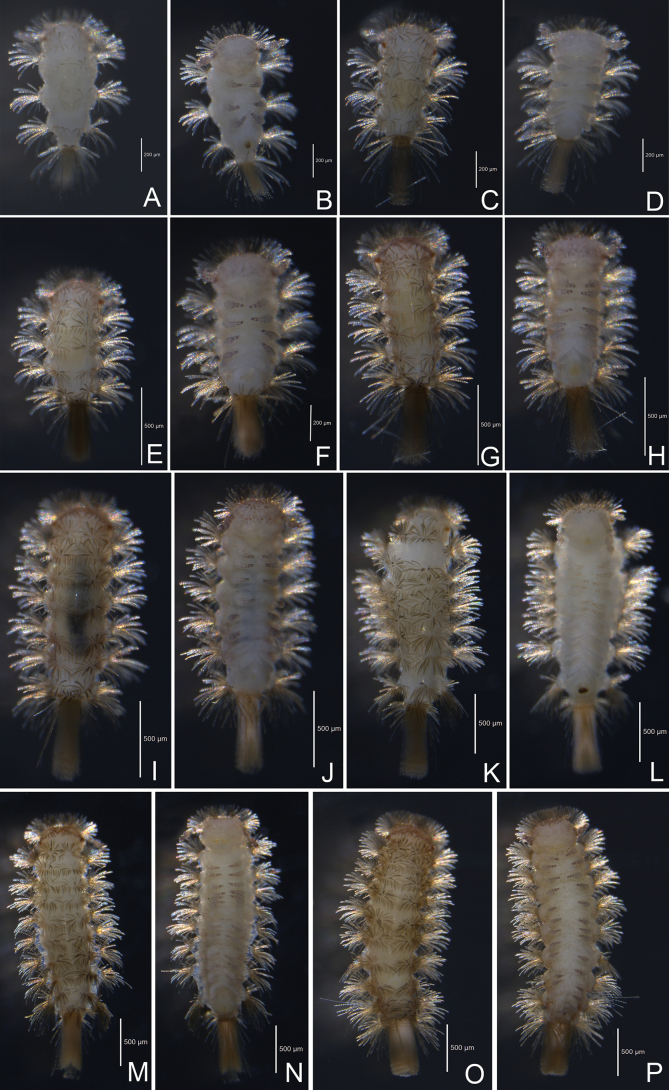
Habitus of post-embryonic development stadia of *Eudigraphishuadongensis* sp. nov. **A, B.** Stadium I; **C, D.** Stadium II; **E, F.** Stadium III; **G, H.** Stadium IV; **I, J.** Stadium V; **K, L.** Stadium VI; **M, N.** Stadium VII **O, P.** Adult.

Specimens of each stadium were mounted and observed under a microscope. The results indicate that the post-embryonic development of the present species is similar to *E.taiwaniensis* (Ishii, 1990), with seven juvenile stadia observed (Fig. 9). Compared with adults, juveniles have small body size, fewer ommatidia, tergites, legs and trichomes, shorter antenna and absence of some sensilla on antennae and lateral palp of gnathochilarium (Table 5). Sex organs first present in stadium VII, but with fewer setae, three on upper plate and 3+3 on lower plates in female and six in male. The morphological characters of each stadium are given in Table 5.

**Table 5.** Morphology of each stadium and adults of *Eudigraphishuadongensis* sp. nov.

Stadium	I	II	III	IV	V	VI	VII	Adult
**Body length (mm)**	0.7–0.8	1.0–1.2	1.3–1.7	1.7–1.8	2.0–2.1	2.1–2.5	2.9–3.2	3.0–3.6
**Head length (μm)**	150–185	190–200	200–250	210–250	250–325	250	250–305	270–450
**Head width (μm)**	260–290	285–330	300–360	300–375	325–400	425–450	475–500	400–600
**Antenna length (μm)**	225–250	225–250	260–300	275–325	300–375	350–400	350–400	400–500
**Leg pairs**	3	4	5	6	8	10	12	13
**Number of tergites**	4	4	5	6	7	8	9	10
**Number of ommatidia**	5	5	7	8	8	8	8	8
**Antennal articles**	5	5	7	8	8	8	8	8
**Antennal sensilla**	lack Ta on article III; article IV same as adult	lack Ta on article III; article IV same as adult	same number as adult, but with short Ta on article V; article VI as adult	Ta become longer on article VI; article VII as adult	same as adult	same as adult	same as adult	article VI with Ta, s, Ti, Tp, c; article VII with Ta, s, Tp, c
**setae of Clypeo-labrum**	4+4	4+4	4+4	4+4	4+4	4+4 or 5+5	4+4 or 5+5	5+5, sometimes 4+4
**Number of sensilla on medial palp of gnathochilarium**	20 (21)	20 (21)	20 (21)	20 (21)	20 (21)	20 (21)	20 (21)	20 (21)
**Number of sensilla on lateral palp of gnathochilarium**	10	10	10 (12)	12	12	12	12	12
**Number of trichomes on posterior vertex per side**	a-row: 6–7; p-row: 3–4	a-row: 7–8; p-row: 3–4	a-row: 8–9; p-row: 4–5	a-row: 8–10; p-row: 4–6	a-row: 8–11; p-row: 5–9	a-row: 10–11; p-row: 6–9	a-row: 11–14; p-row: 7–8	a-row: 11–16; p-row: 7–13
**Number of trichomes on collum per side**	10–14	13–17	19–20	21–27	30–33	31–39	34–41	43–52
**Number of trichomes on lateral protuberance of collum**	2–3	2–4	3	3–4	4	4–5	4–5	5–6
**Number of dorsal ornamental trichomes per side**	5–6 a, b, c1, c3	7–10 a, b, c1, c3	8–10 a, b, c1, c2, c3	10–14 a, b, c1, c2, c3	8–14 a, b, c1, c2, c3	11–17 a, b, c1, c2, c3	13–15 a, b, c1, c2, c3	11–17 a , b, c1, c2, c3,
**Specimens examined**	7	6	7	8	6	3	4	45

### ﻿Mitogenome structure and organization

**Figure 10.**
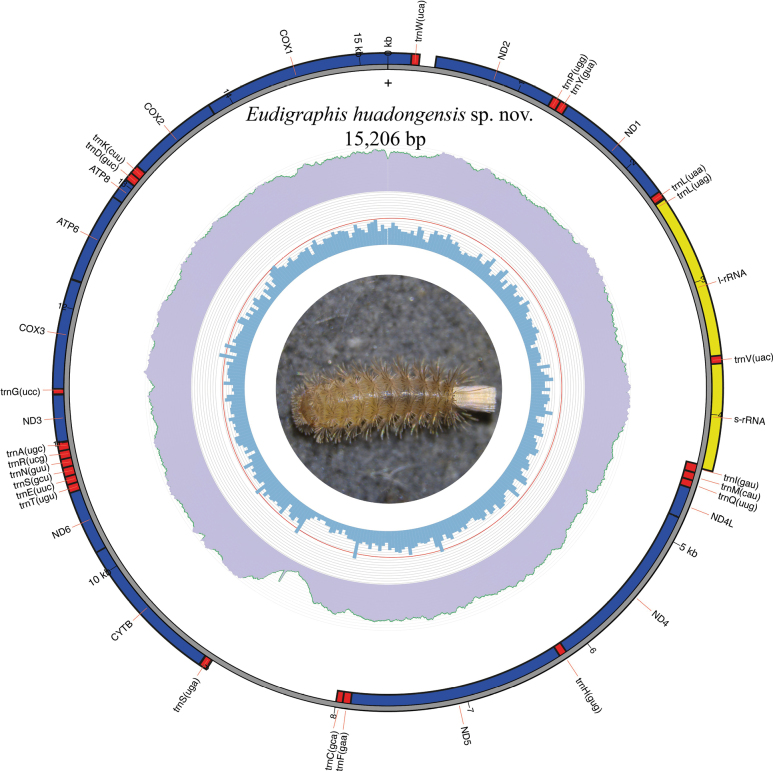
Circular representation of the mitogenome of *Eudigraphishuadongensis* sp. nov. The innermost circle shows the GC content; the middle circle shows the reads coverage, and the outermost circle shows the gene features, rRNA (yellow), tRNA (red), and CDS (dark blue). The photo in the center represents a live specimen.

**Figure 11.**
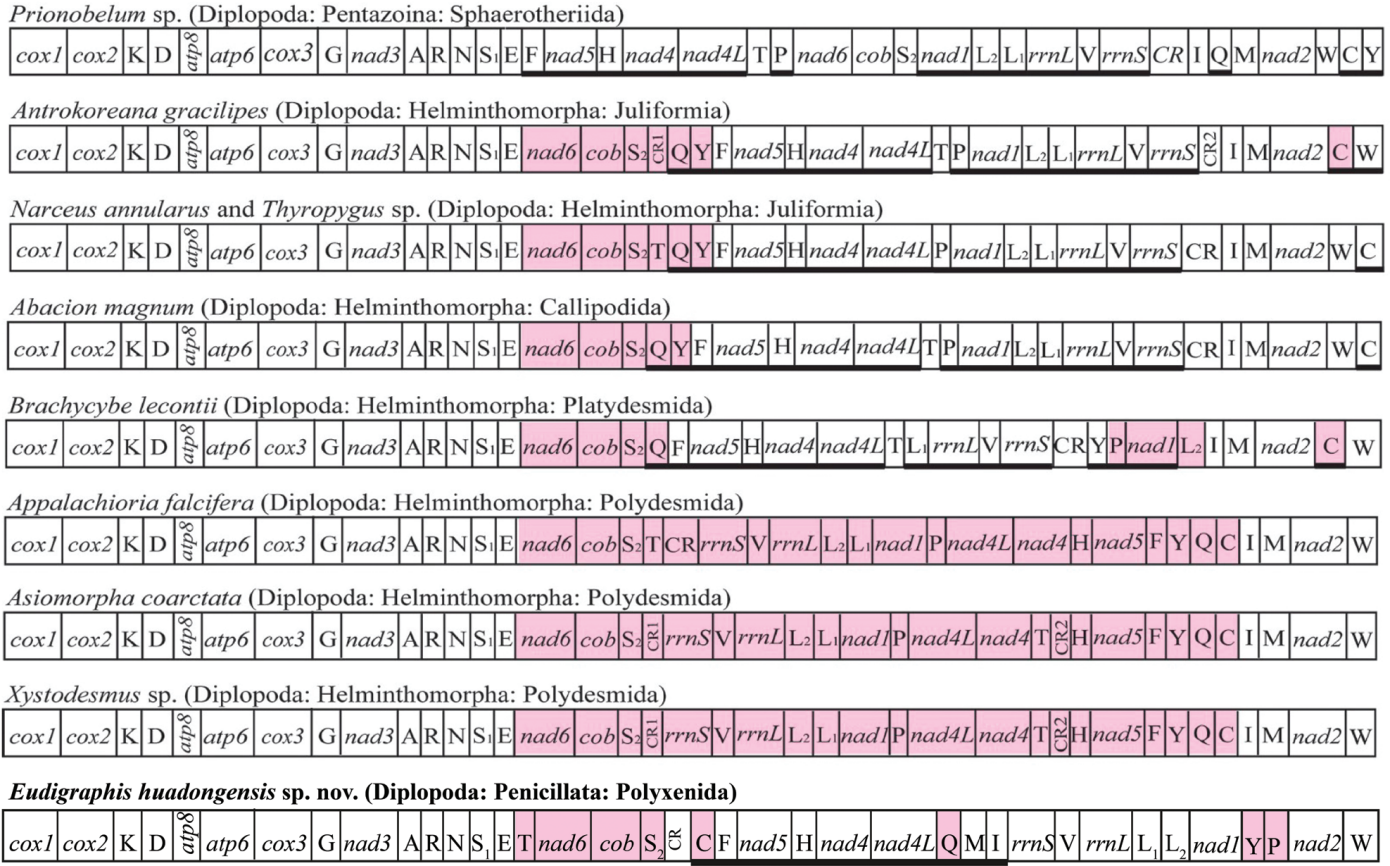
Comparison of gene arrangements in mtDNA of nine Diplopoda species, including *Eudigraphishuadongensis* sp. nov. (in bold). *Prionobelum* sp. shares the ancestral arthropod ground pattern. Gene segments are not drawn to scale. Genes highlighted in pink have different relative positions compared to the ancestral arthropod ground pattern. Underlining indicates the gene is encoded on the opposite strand. CR: putative control region. (Figure adapted from Dong et al. 2016).

The mitogenome of *Eudigraphishuadongensis* sp. nov. is a circular molecule of 15,206 bp in length. All typical 13 protein-coding genes, 22 tRNAs, two ribosomal RNA genes, and one control region were identified (Fig. 10). The genes between tRNA-Ile (trnI) and tRNA-Cys (trnC) are encoded on the heavy (+) chain, while the remaining genes are encoded on the light (–) chain. The gene order of the new mitogenome is unique when compared to other Diplopoda species, and to the Arthropod ancestral gene order (AGO) shared by *Prionobelum* sp., a representative of the Diplopoda class (Dong et al. 2012, 2016) (Fig. 11). The region located between NAD6 and tRNA-Trp (trnW) is highly variable in all analyzed mitogenomes, and the region between COX1 and tRNA-Glu (trnE) is conserved (Fig. 11). The putative control region of *E.huadongensis* sp. nov. is 987 bp long and is located after tRNA-Ser (trnS_2_), same arrangement as three of the Diplopoda mitogenomes analyzed here, but different from AGO (Fig. 11). After the control region, the tRNA-Cys (trnC) was translocated, this location is exclusive for the newly assembled mitogenome. The most common initiation codes were ATT and ATG, and the TAA and TAG were the most used termination codes. NAD2, NAD4, NAD5 have incomplete stop codons (Table 6).

**Table 6.** Features of the mitochondrial genome of *Eudigraphishuadongensis* sp. nov. The direction of coding strands is indicated by major strand (+) and minor strand (-).

*Eudigraphishuadongensis* sp. nov. mitogenome length 15,206 bp						
Start	End	Length (bp)	Direction	Type	Gene name	Start-stop codons
0	171	171	-	CDS	COX1	ATG – TAA
171	232	62	-	tRNA	trnW(uca)	
352	1250	899	-	CDS	ND2	ATA – T--
1250	1311	62	-	tRNA	trnP(ugg)	
1312	1374	63	-	tRNA	trnY(gua)	
1373	2291	919	-	CDS	ND1	TTG – TAG
2285	2344	60	-	tRNA	trnL(uaa)	
2344	2404	61	-	tRNA	trnL(uag)	
2350	3737	1388	-	rRNA	l-rRNA	
3566	3628	63	-	tRNA	trnV(uac)	
3624	4413	790	-	rRNA	s-rRNA	
4386	4455	70	+	tRNA	trnI(gau)	
4456	4517	62	+	tRNA	trnM(cau)	
4517	4576	60	+	tRNA	trnQ(uug)	
4576	4852	277	+	CDS	ND4L	ATT – TAA
4817	6189	1373	+	CDS	ND4	ATG – ---
6165	6225	61	+	tRNA	trnH(gug)	
6225	7916	1692	+	CDS	ND5	ATT – TA-
7888	7950	63	+	tRNA	trnF(gaa)	
7950	8001	52	+	tRNA	trnC(gca)	
8002	8989	987			Control region	
8990	9050	61	-	tRNA	trnS(uga)	
9053	10178	1126	-	CDS	CYTB	ATG – TAA
10161	10638	478	-	CDS	ND6	GTG – TAA
10632	10694	63	-	tRNA	trnT(ugu)	
10694	10754	61	-	tRNA	trnE(uuc)	
10754	10821	68	-	tRNA	trnS(gcu)	
10821	10884	64	-	tRNA	trnN(guu)	
10883	10946	64	-	tRNA	trnR(ucg)	
10945	11011	67	-	tRNA	trnA(ugc)	
11009	11357	349	-	CDS	ND3	ATT – TAG
11360	11422	63	-	tRNA	trnG(ucc)	
11399	12206	808	-	CDS	COX3	ATG – TAA
12209	12884	676	-	CDS	ATP6	ATG – TAA
12877	13033	157	-	CDS	ATP8	GTG – TAG
13033	13096	64	-	tRNA	trnD(guc)	
13094	13160	67	-	tRNA	trnK(cuu)	
13160	13841	682	-	CDS	COX2	ATG – TAA
13841	15206	1365	-	CDS	COX1	ATG – TAA

### ﻿Genetic divergence analysis

**Figure 12.**
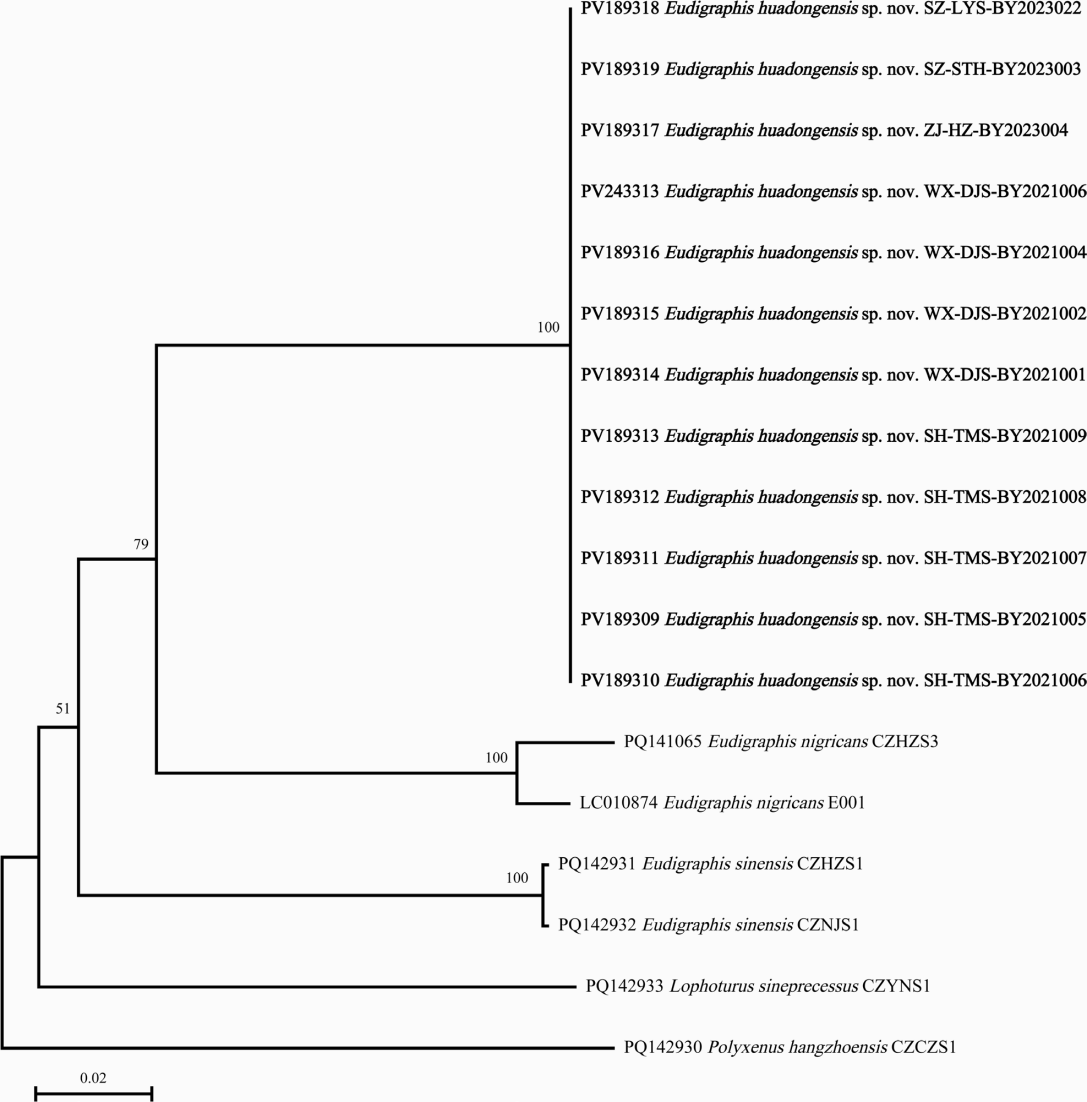
Neighbor-Joining tree (Jukes-Cantor model, Bootstrap 1000 replicates) of *Eudigraphis* spp. constructed by 5’- end sequences data set (642 bp). Numbers at the nodes show the bootstrap values.

**Figure 13.**
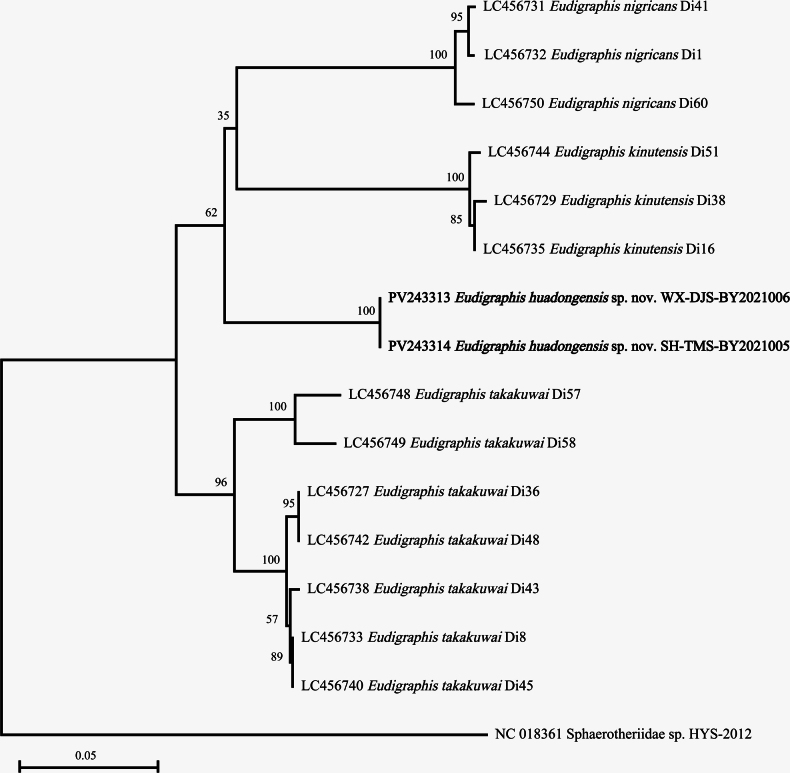
Neighbor-Joining tree (Jukes-Cantor model, Bootstrap 1000 replicates) of *Eudigraphis* spp. tree constructed by 3’- end sequences data set (534 bp). Numbers at the nodes show the bootstrap values.

After alignment of all available COI gene sequences, two data sets were obtained: one close to 5’-end includes 642 base pairs, another one close to 3’-end includes 534 base pairs in total, which were analyzed separately. The results indicated that the COI gene of the new species was fairly conserved among populations: all sequences of individuals collected from five localities were completely the same. The Neighbor-Joining trees supported that *E.huadongensis* sp. nov. is a unique species different from other congeners (Figs 12–13).

The genetic distances between *E.huadongensis* sp. nov. and other congeners are: 0.1624 on average (0.1509–0.1668) for 5’-end fragments, and 0.1323 on average (0.1151–0.1601) for 3’-end fragments, which are distinctly higher than conspecific distances of the genus 0.0269 on average (0–0.0625), and well match the spans of interspecific distances within the genus 0.1552 on average (0.1151–0.1765).

## ﻿Discussion

*Eudigraphishuadongensis* sp. nov. is the fifth Chinese species of this genus and is widely distributed in East China. It lives in the litter, humus, or the upper layer of soil of different kinds of forests, never dwelling under tree bark. Our experience of collection indicated that it prefers a relatively dry environment, with high density in dry litter. It has a cylindrical body that is similar to *E.taiwaniensis*, different to the species under tree bark that have flat bodies such as *E.sinensis* and *E.nigricans*.

After detailed comparison of all known species of the genus, we found that the body color, sensilla on the antennae, structures of the mandible, labrum, sensilla on palps of gnathochilarium, and the shape of setae on legs are good diagnostic characters for species separation (Table 4). Some characters such as sockets on posterior vertex, sockets on tergites, and dorsal ornamental trichomes on the telson are fairly variable and their numbers often overlap between species; they have less taxonomic value and are not ideal characters for differentiating to species level.

There are few molecular data available for Polyxenida and no previous mitochondrial genome has been determined for the order. We sequenced the complete mitochondrial genome of *Eudigraphishuadongensis* sp. nov. and compared it with that of other diplopods. Its gene order is exclusive and new studies are necessary to investigate if the model found here is diagnostic for the Polyxenida group. The genetic distances of COI gene indicated that *E.huadongensis* sp. nov. can be well separated from other congeners, further supporting our morphological identification.

## Supplementary Material

XML Treatment for Eudigraphis

XML Treatment for Eudigraphis huadongensis

## ﻿Additional information

### Use of AI

No use of AI was reported.

### Author ORCIDs

Si-Qi Yang https://orcid.org/0009-0000-4331-792X

Yun Bu https://orcid.org/0000-0002-7177-9686

Nerivania Nunes Godeiro https://orcid.org/0000-0002-1669-6124

Yan Gao https://orcid.org/0000-0003-0305-3696

Ya-Li Jin https://orcid.org/0009-0003-5773-2165

### Data availability

All of the data that support the findings of this study are available in the main text.
